# Genomic Profiling of Chinese Cervical Cancer Patients Reveals Prevalence of DNA Damage Repair Gene Alterations and Related Hypoxia Feature

**DOI:** 10.3389/fonc.2021.792003

**Published:** 2022-01-07

**Authors:** Hao Wen, Qin-Hao Guo, Xiao-Lan Zhou, Xiao-Hua Wu, Jin Li

**Affiliations:** ^1^ Department of Oncology, Shanghai Medical College, Fudan University, Shanghai, China; ^2^ Department of Gynecologic Oncology, Fudan University Shanghai Cancer Center, Fudan University, Shanghai, China; ^3^ Hua-Shan Worldwide Medical Center, Hua-Shan Hospital, Fudan University, Shanghai, China

**Keywords:** cervical cancer, Chinese cohort, Western cohort, genomic alterations, actionable alterations, DDR, hypoxia, tumor microenvironment

## Abstract

**Background:**

Cervical cancer is responsible for 10–15% of cancer-related deaths in women worldwide. In China, it is the most common cancer in the female genital tract. However, the genomic profiles of Chinese cervical cancer patients remain unclear.

**Materials and Methods:**

A total of 129 cervical cancer patients were enrolled in this study (113 squamous, 12 adenocarcinoma, 2 adenosquamous, and 2 neuroendocrine carcinoma). To classify the clinical features and molecular characteristics of cervical cancer, the genomic alterations of 618 selected genes were analyzed in the samples of these patients, utilizing target next-generation sequencing (NGS) technology. Furthermore, the findings from the Chinese cohort were then compared with the data of Western patients downloaded from The Cancer Genome Atlas (TCGA) database, in terms of gene expression files, mutation data, and clinical information.

**Results:**

All studied patients had valid somatic gene alterations, and the most frequently altered genes were *PIK3C*, *TP53*, *FBXW7*, *ARID1A*, *ERBB2*, and *PTEN*. Comparison of genomic profiling showed significantly different prevalence of genes, including *TP53, KMT2C*, and *RET*, between the Chinese and the TCGA cohorts. Moreover, 57 patients (44.19%) with 83 actionable alterations were identified in our cohort, especially in PI3K and DNA damage repair (DDR) pathways. After an in-depth analysis of cervical cancer data from the TCGA cohort, DDR alteration was found to be associated with extremely higher tumor mutation burden (TMB) (median mutation count: 149.5 vs 66, p <0.0001), and advanced stages (p <0.05). Additionally, DDR alteration, regardless of its function, was positively correlated with hypoxia feature and score. Moreover, patients with a high hypoxia score were positively correlated with a high abundance of mast cell resting, but lower abundance of CD8+ T cells and activated mast cell. Finally, *CDHR5* was identified as the hub gene to be involved in the DDR–hypoxia network, which was negatively correlated with both the DDR alteration and hypoxia score.

**Conclusions:**

Overall, a unique genomic profiling of Chinese patients with cervical cancer was uncovered. Besides, the prevalent actionable variants, especially in PI3K and DDR pathways, would help promote the clinical management. Moreover, DDR alteration exerted the significant influence on the tumor microenvironment in cervical cancer, which could guide the clinical decisions for the treatment. *CDHR5* was the first identified hub gene to be negatively correlated with DDR or hypoxia in cervical cancer, which had potential effects on the treatment of immune checkpoint inhibitors (ICIs).

## Introduction

Cervical cancer is the fourth most common cancer among women worldwide, affecting nearly 600,000 women annually (1). The application of the human papillomavirus (HPV) vaccine and screening programs have significantly reduced the incidence of cervical cancer; however, it is still highly prevalent in developing countries as the second most common cause of cancer-related deaths in women (2). Even though the disease at its early stages can be amenable to surgery or radiotherapy, recurrent or metastatic cervical cancer is still incurable and calls for novel therapeutic approaches (3). In the past decade, the ICI, pembrolizumab, was the only novel treatment approved by the FDA for treating PD-L1-positive, recurrent or metastatic cervical cancer patients with disease progressing on or after chemotherapy. Though it offers new hope for advanced disease, it is notable that its efficacy was still poorly limited, with an objective response rate of 14.6% in patients with PD-L1–positive tumors (4). Thus, a better understanding of the genomic feature of cervical cancer is a fundamental part for the identification of biomarkers for the development of novel therapeutic approaches and improvement of the efficacy of ICIs.

With the great advances of next-generation sequencing, it enables the researchers to find a comprehensive genomic feature and identify the treatment-related biomarkers in cervical cancer patients. The genomic profiles of Western patients with cervical cancer have been revealed by the TCGA project in 2017 (5). A high prevalence of genes, namely, *PIK3CA*, *EP300*, *FBXW7*, and *PTEN* was identified as the genomic feature of Western cervical cancer patients, and inferred as novel potential therapeutic targets for drug development in future. Meanwhile, in a pan-cancer study, researchers from the Memorial Sloan-Kettering Cancer Center (MSKCC) uncovered that over one third of metastatic cervical cancer patients harbored at least one actionable alteration (6). However, comparing with other tumor types, the number of studies in genetic profiling on cervical cancer is relatively limited. Furthermore, previous studies were predominantly on the Caucasian patients, leaving an unsolved question on whether there were genetic differences between Chinese and Western cervical patients.

Recently, it has been found that DDR alteration(s) could influence the inflammatory signaling pathways which have the ability of reshaping tumor microenvironment (7), and are emerging as an effective biomarker for predicting the response of ICI, for example, (1) DDR alterations were significantly correlated with clinical benefit in urothelial carcinoma patients who received the therapeutic treatment of anti-PD1/PDL1 (8), (2) ICIs therapy could improve the survival of non-small cell lung cancer patients having co-mutations of DNA damage response and repair pathways (9), and (3) DDR mutations were correlated with improved overall survival of patients with colorectal cancer (10). Meanwhile, it has been comprehensively studied in other gynecological tumors, especially in ovarian and endometrial carcinoma as the hallmark event for precision medicine or prognosis classification. However, the role of DDR in cervical cancer has not been specifically clarified yet. In cervical cancer, HPV could manipulate DDR genes to improve its viral life and prevent the viral apoptosis (11). In addition, the progression of cervical cancer is significantly associated with the increased genetic instability, which is primarily caused by the abnormal regulation of DDR genes (12). Thus, the latest Clinical Trials Planning Meeting from the National Cancer Institute (NCI) in 2020 have stressed the development of clinical trials to explore the potential role of DDR in the treatment of cervical cancer (13).

To our knowledge, there existed several studies describing genomic features of Chinese cervical cancer patients, namely, 13 cervical cancer cases of Chinese Hong Kong women (14), 20 endocervical adenocarcinoma cases (15), 32 cervical cancer cases (16), 32 advanced cervical cancer (17), and 74 cervical cancer cases (18) of Chinese mainland women. However, the latter two studies focused on the molecular profiles of integrated gynecologic cancers containing ovarian cancer, endometrial cancer, and cervical cancer. In the present study, we performed the NGS to determine the genomic profiling of 129 Chinese cervical cancer patients, especially the actionable alterations to explore some potential therapeutic strategies. Furthermore, by comparison with the data from Western cohort, it was the first time to figure out the genetic difference(s) between Chinese and Western patients with cervical cancer. Subsequently, we further explored the DDR alteration and tumor microenvironment based on the public dataset.

## Materials and Methods

### Sample Source and Ethic Data

A total of 129 cervical cancer patients were enrolled in the Fudan University Shanghai Cancer Center, from 2018 to 2020. A total of 72 of enrolled patients (55.81%) had sufficient achieved tumor tissues, while the rest provided blood samples instead for genetic testing, mostly for the following reasons: (i) tumor samples were pathologically reviewed and having tumor cells less than 20%; (ii) no valid or sufficient archived tumor tissue samples; and (iii) diagnosed as metastatic or recurrent disease and more willing to have liquid biopsy testing to exclude potential heterogeneity. Blood samples were drawn into Streck Cell-Free DNA collection tubes and stored at 4°C. Demographics and clinical data were collected for analysis. All patients had provided with signed informed consent and agreed to publish related genomic data without revealing personal identity.

### DNA Isolation and Targeted Next-Generation Sequencing

Genomic DNA (gDNA) of tumor samples and germline DNA (from white blood cells) were isolated using QIAamp DNA FFPE Tissue Kit (Qiagen, CA, USA) according to the manufacturer’s instruction. Circulating cell-free DNA (cfDNA) was extracted using a QIAamp Circulating Nucleic Acid Kit (Qiagen, CA, USA). Quantity and quality of the purified DNA were checked using Qubit 2.0 Fluorometer (Thermo Fisher Scientific, MA, USA) and Bioanalyzer 2100 (Agilent Technologies, CA, USA). A total of 100 ng of gDNA was sheared with a Covaris E210 system (Covaris, MA, USA) to target fragment sizes of 200 bp. We performed library preparation for tumor gDNA (>30 ng), cfDNA (>20 ng) and matched germline gDNA (>100 ng) using Accel-NGS 2S DNA Library Kit (Swift Biosciences, MI, USA) and target enrichment using xGen Lockdown Probes kit (Integrated Device Technology, Inc., CA, USA). The custom xGen Lockdown probe was synthesized by IDT, Inc. for the exons and parts of introns of 618 genes of interest. Samples underwent paired-end sequencing on an Illumina Novaseq 6000 platform (Illumina, CA, USA) with a 150-bp read length. The minimum coverage of 1,000×, 3,000×, and 500× were achieved for tumor gDNA, plasma cfDNA, and germline DNA, respectively.

### Database and Genomic Analysis

Raw sequencing data were aligned to the reference human genome (UCSC hg19) through Burrows–Wheeler Aligner and producing a binary alignment/map (BAM) file. After the duplicate removal and local realignment by using Picard (http://broadinstitute.github.io/picard/), the Genome Analysis Toolkit (GATK) was used for single nucleotide variation (SNV), short insertions/deletions (indels) calling. Variants were annotated using the ANNOVAR software tool. Variants identified in gDNA from white blood cell (WBC) with allele fraction (AF) beyond 25% were determined as germline variants. Germline variants were filtered with following rules: (i) allele frequency (AF) below 25%; (2) variants were synonymous or not in the coding region (not including the splice-site variants); (3) occurred in over 1% population in the ExAC database (http://exac.broadinstitute.org/); and (4) known benign or likely benign variants (Clinvar). Interpretation of germline variants followed the standards and guidelines of the American College of Medical Genetics and Genomics and the Association for Molecular Pathology (ACMG/AMP).

After filtering out the germline variants identified in the matched WBC samples, variants with allele frequency (AF) beyond 1% were generated from each tumor gDNA and AF beyond 0.5% for plasma cfDNA, and further annotated according to the Catalog of Somatic Mutations in Cancer (COSMIC) database. The functional classification of each somatic alteration followed the interpretation and reporting standards and guidelines recommended by the Association for Molecular Pathology, American Society of Clinical Oncology, and College of American Pathologists (ASCO/CAP) and the Oncokb database (through cBioPortal for Cancer Genomics at http://www.cbioportal.org/) (19). Somatic mutation data, gene expression profiles, and clinical information of cervical cancer patients from the TCGA cohort were downloaded from the cBioPortal.

### Analysis of the Functional Enrichment, Hypoxia Feature, and Tumor Environment

The “limma” package was used to screen the differentially expressed genes (DEGs) in the two groups using False Discovery Rate (FDR) <0.05 and Fold Change (FC) >1.5. Heatmaps were visualized using the “pheatmap” package. The Gene Ontology (GO) and the Kyoto Encyclopedia of Genes and Genomes (KEGG) pathway enrichment analyses were conducted by using the “ClusterProfiler” package (20) in the R studio (v. 3.4.3, https://rstudio.com/). The tumor mutation burden of each sample was calculated according to a published and widely applied method (21). The hypoxia feature was quantified by the previously described buffa hypoxia score (22) and ragnum hypoxia score (23). The CIBERSORT algorithm was used to calculate the proportion of infiltrating immune cells in cervical cancer samples (24).

### Statistical Analysis

Differential mutations analysis was performed using the Chi-Square test or Fisher exact test under a dominant model. Two-sided P values less than 0.05 were considered to be statistically significant. All analyses were performed using SPSS 25.0 software.

## Results

### The Characteristics of Cervical Cancer Patients in the Chinese Cohort

One hundred and twenty-nine Chinese patients diagnosed with cervical cancer were enrolled in this study with a median age of 48 (n = 97, range: 21 to 78 years). The subtypes included squamous cell carcinoma (SCC, n = 113, 87.60%), adenocarcinoma (AC, n = 12, 9.30%), adenosquamous carcinomas (ASC, n = 2, 1.55%), and neuroendocrine carcinoma (NEC, n = 2, 1.55%). In addition, 75 of the patients (58.14%) have diseases of FIGO (International Federation of Gynecology and Obstetrics [FIGO] staging system) stages III–IV (**Table 1**).

**Table 1:** Characteristics of 129 patients with cervical cancer.

Variables		n (%)
Total		129
Age	Mean (range)	48 (21–78)
Histologic type		
	Squamous cell	113 (87.60%)
	Adenocarcinoma	12 (9.30%)
	Adenosquamous	2 (1.55%)
	Neuroendocrine	2 (1.55%)
FIGO Stage		
	I	11 (8.53%)
	II	43 (33.33%)
	III	38 (29.46%)
	IV	37 (28.68%)
Sample Type	Blood	57 (44.19%)
	Tumor	72 (55.81%)

FIGO stage, International Federation of Gynecology and Obstetrics [FIGO] staging system.

### Somatic and Germline Alterations in Chinese Cervical Cancer Patients, and Correlation Between Genomic Alterations and Histologic Types

**Figure 1:**
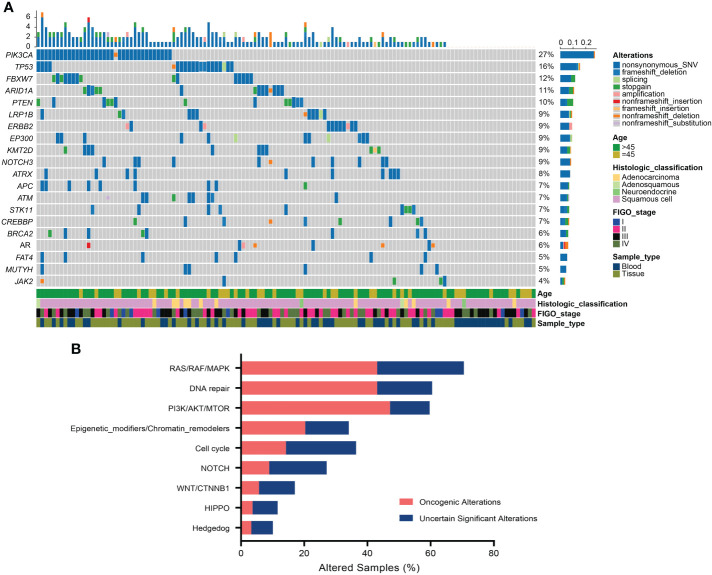
Somatic alterations in Chinese cervical cancer patients. **(A)** Oncoprint of the top 20 frequently altered genes in 129 cervical cancer patients. **(B)** The distribution of most recurrent altered signaling pathways in the Chinese cervical cancer.

All surveyed samples had been identified with valid somatic alterations, and the mean and median counts of somatic alterations per sample were 8.35 and 7, respectively. The most frequently altered genes in the patients were *PIK3CA* (27.13%), *TP53* (15.50%), *FBXW7* (11.63%), *ARID1A* (10.85%), and *PTEN* (10.08%), respectively (**Figure 1A**). We noticed that 9.30% of patients had *ERBB2* alterations and 6.97% of those had oncogenic alterations which were only identified in the tissue samples. Additionally, the most recurrent altered signaling pathways included RAS/RAF/MAPK (70.54%), DDR (60.47%), PI3K/ATK/MTOR (59.69%), cell cycle (36.43%), and epigenetic modifiers/chromatin remodelers (34.11%) (**Figure 1B**). Of note, PI3K/ATK/MTOR pathway had the most oncogenic alterations (47.15%). Moreover, three patients (2.33%) harbored pathogenic or likely pathogenic germline variants, including one *ATR*-K704*, one *BRCA1*-S1841fs, and one *POLE*- S2173fs, respectively.

In addition, the correlation analysis was further conducted to investigate whether one specific histologic subtype of cervical cancer was associated with the most frequently altered genes. It was found that there was a statistically significant difference in the alteration frequency of *TP53* among these four histologic types, showing that *TP53* alteration happened more frequently in ACs and ASCs (p = 0.003, **Table 2**). Moreover, it could be obviously observed that all *ARID1A* alterations happened in SCCs, but with no statistically significant difference (p = 0.214, **Table 2**).

**Table 2:** Genomic alterations in four histologic types among 129 cervical patients.

Altered gene (Patient number)	Histologic type				p-value
	SCC^1^	AC^2^	ASC^3^	NEC^4^	
	n = 113	n = 12	n = 2	n = 2	
*PIK3CA* (n = 35)	33 (29.20%)	1 (8.33%)	1 (50.00%)	0 (0.00%)	0.232
*TP53* (n = 20)	13 (11.50%)	6 (50.00%)	1 (50.00%)	0 (0.00%)	0.003
*FBXW7* (n = 15)	13 (11.50%)	2 (16.67%)	0 (0.00%)	0 (0.00%)	1.000
*ARID1A* (n = 14)	14 (12.39%)	0 (0.00%)	0 (0.00%)	0 (0.00%)	0.214
*PTEN* (n = 13)	10 (8.85%)	1 (8.33%)	1 (50.00%)	1 (50.00%)	0.205

^1^Squamous cell carcinoma.

^2^Adenocarcinoma.

^3^Adenosquamous carcinoma.

^4^Neuroendocrine carcinoma.

### The Comparison of Genomic and Actionable Alterations of Cervical Cancer Patients Between Chinese Cohort and Western Cohort

**Figure 2:**
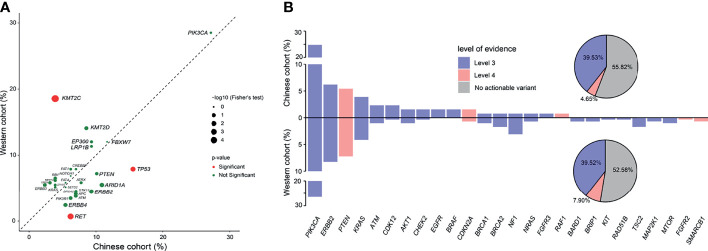
Comparison of genomic and actionable alterations of cervical cancer patients between the Chinese cohort and the TCGA cohort. **(A)** Comparison of the prevalence of gene alterations identified between Chinese and Western cervical cancer patients. **(B)** Comparison of the actionable alterations identified between Chinese and Western cervical cancer patients.

To determine the potential differences of genomic feature between Chinese and Western cervical cancer patients, we conducted a comparison of the genomic alterations data of the selected 618 genes between the Chinese and the Western cohort (published by the TCGA project) to identify the genetic differences. The genomic feature between the Chinese and the Western cohorts was similar, except the significant different prevalence of alterations in *KMT2C* (Chinese cohort vs Western cohort: 3.88% vs 18.56%), *RET* (Chinese cohort vs Western cohort: 6.20% vs 0.69%), and *TP53* (Chinese cohort vs Western cohort: 15.50% vs 7.90%) (p <0.05, **Figure 2A**).

Next, we compared the frequency of actionable alterations between the Chinese and Western cohorts. Based on the OncoKB Levels of Evidence V2 (12/20/2019), 57 patients (44.19%) with 73 actionable alterations were identified in the Chinese cohort (**Figure 2B**), of which the ratio was approximately similar to the prevalence of actionable alterations in the Western cohort (47.42%). Besides, more patients had actionable variants of level 3 than level 4 (39.53% vs 4.65%), as nearly a quarter of the cervical cancer patients had actionable of alterations in *PIK3CA*, which may confer sensitivity to the PI3K or mTOR inhibitors. The rest actionable alterations were mainly enriched in the DDR and RAS/RAF/MAPK pathways, associated with increasing sensitivity to the poly (ADP-ribose) polymerase (PARP) Inhibitors and receptor tyrosine kinases (RTKs) inhibitors.

### Alterations in DNA Damage Repair Pathway

**Figure 3:**
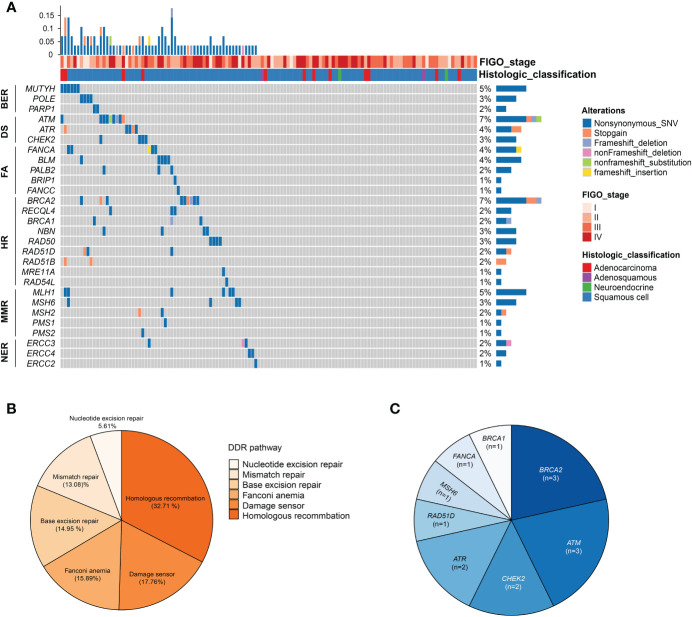
Alterations in DNA damage repair (DDR) pathway. **(A)** Oncoprint of the DDR alterations in 129 cervical cancer patients. **(B)** Frequency of altered pathway of DDR. **(C)** The distribution of known or likely deleterious DDR alterations. HR, homologous recombination; FA, fanconi anemia; MMR, mismatch repair; NER, nucleotide excision repair; BER, base excision repair; DS, DNA sensor.

A total of 61 patients (47.29%) harbored at least one alteration in 34 DNA repair genes defined by MSKCC (25), and the prevalence of specific genes in DDR was exhibited in **Figure 3A**. In addition, the frequently altered DDR signaling pathways were Homologous recombination (32.71%), Damage sensor (17.76%), Fanconi anemia (15.89%), Base excision repair (14.95%), Mismatch repair (13.08%), and Nucleotide excision repair (5.61%) (**Figure 3B**). Genes with known or likely deleterious variants among cervical cancer patients with DDR gene alterations were *ATM* (n = 3, 2.33%), *BRCA2* (n = 3, 2.33%), *ATR* (n = 2, 1.55%), *CHEK2* (n = 2, 1.55%), followed by *BRCA1* (n = 1, 0.78%), *FANCA* (n = 1, 0.78%), *MSH6* (n = 1, 0.78%), and *RAD51D* (n = 1, 0.78%) (**Figure 3C**).

### The Comparison of Clinical Features of Cervical Cancer Patients With or Without DDR Alteration(s)

**Figure 4:**
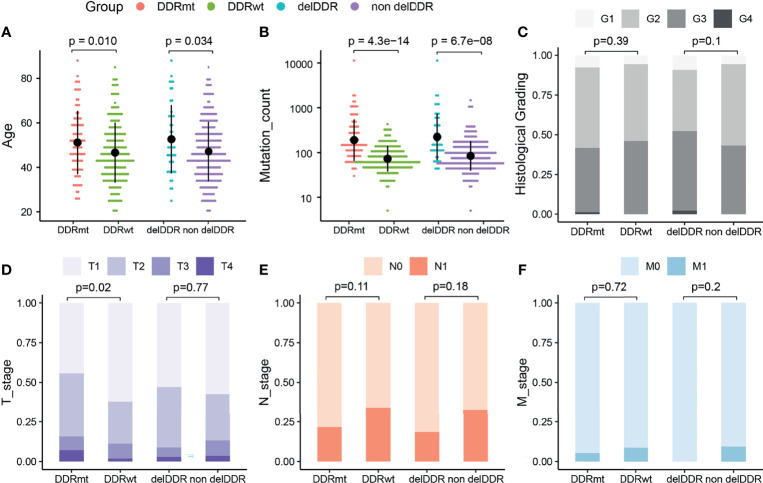
The analyses of clinical features of cervical cancer patients with DDR and DDR alteration from the TCGA cohort. **(A)** Age at diagnosis of cervical cancer patients with and without any DDR alteration, and patients with deleterious DDR alteration or not. **(B)** Mutation count of cervical cancer patients with and without any DDR alteration, and patients with deleterious DDR alteration or not. Histological grading **(C)**, tumor stage **(D)**, lymph node stage **(E)**, and metastasis stage **(F)** of cervical cancer patients with and without any DDR alteration, and patients with deleterious DDR alteration or not. DDRmt, patients with any DDR alteration; DDRwt, patients without any DDR alteration; delDDRmt, patients with deleterious DDR alteration; nondelDDRmt, patients without any deleterious DDR alteration.

We identified a total of 92 cervical cancer patients (31.62%) from the TCGA cohort harboring DDR alterations, including 47 (16.15%) and 45 (15.46%) patients having deleterious DDR alteration and non-deleterious DDR alteration, respectively. The prevalence of total DDR alterations in the Western cohort was significantly lower than the Chinese cohort (p <0.05). Next, we investigated the clinical features of cervical cancer patients with any DDR alteration (DDRmt group, N = 92) and without DDR alteration (DDRwt group, N = 199). Interestingly, a significantly older age at diagnosis was observed in the DDRmt group (average age at diagnosis: 51.18 vs 46.64 years old, p = 0.01, **Figure 4A**), and also more genetic mutations (median mutation count: 149.5 vs 66, p <0.0001, **Figure 4B**). However, according to the histological grading for cervical cancer, there was no statistically significant difference between the two groups (DDRmt vs DDRwt group, G1–G2: 58.22% vs 53.88%, G3–G4: 41.78% vs 46.12%, p = 0.39, **Figure 4C**). Besides, we found a significantly decreased number of patients with T1 stage disease but a significantly increased patient number at T2 or T4 stage in the DDRmt group (stage T1: 44.29% vs 62.11%; stage T2: 40.00% vs 26.71%, p <0.05; stage T3: 8.57% vs 9.31% at T3; stage T4:7.14% vs 1.86%, p <0.05, **Figure 4D**). Also, no significant correlation between DDR mutation and the lymph node metastasis or long-distance metastasis status was found (p >0.05, **Figures 4E, F**).

In addition, we further explored the clinical features of the groups with deleterious DDR alteration (N = 47) or without (N = 244) this genomic feature. Similar to patients with any DDR alteration, we identified a significantly higher age at diagnosis in the patients with deleterious DDR alteration (average age at diagnosis: 52.66 vs 47.16 years old, p = 0.034, **Figure 4A**), and also a higher mutation count (median mutation count: 149 vs 78, p <0.0001, **Figure 4B**). By the statistical analysis of the patient number in high or low histological grading, we found no significant difference between two groups (p = 0.10, **Figure 4C**). Furthermore, we surveyed the specific associations between TNM stages and cervical cancer patients with deleterious DDR alteration but found neither tumor, lymph node nor long distant metastasis stage was significantly associated with deleterious DDR alteration (p >0.05, **Figures 4E, F**).

### DDR Alteration, Hypoxia Feature, and Tumor Microenvironment

**Figure 5:**
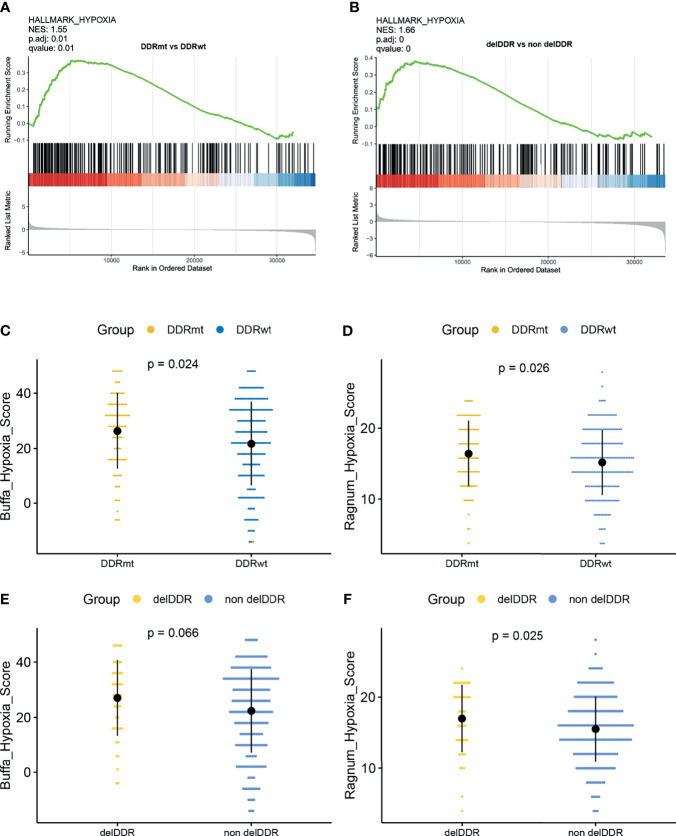
The correlation analyses between the DDR alterations and hypoxia features. **(A)** Gene set enrichment analysis identified hallmark_hypoxia in patients with DDR alteration. **(B)** Gene set enrichment analysis identified hallmark_hypoxia in patients with deleterious DDR alterations. Comparison of the buffa_hypoxia score **(C)** and ragnum_hypoxia score **(D)** between patients with and without any DDR alteration. Comparison of the buffa_hypoxia score **(E)** and ragnum_hypoxia score **(F)** between patients with or without deleterious DDR alterations.

Signaling pathway analysis found that DDR alteration, regardless of its function, was significantly associated with hypoxia feature (**Figures 5A, B**). Subsequently, we found a significant difference in the hypoxia score between patients with or without DDR alteration(s). Remarkably, there was a significantly higher buffa hypoxia score in the DDRmt group (buffa hypoxia score: 26.32 vs 21.70, p = 0.024; ragnum hypoxia score: 16.61 vs 15.34, p = 0.026, **Figures 5C, D**). The findings were concordant when we compared this feature between cervical cancer patients with or without deleterious DDR alteration (buffa hypoxia score: 27.04 vs 22.36; ragnum hypoxia score 17.00 vs 15.49, p = 0.025, **Figures 5E, F**).

**Figure 6:**
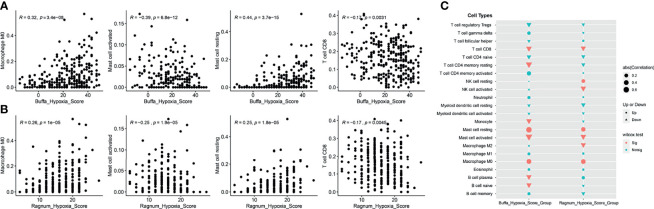
The analysis of tumor microenvironment (TME). The correlation analyses between the buffa **(A)** or ragnum **(B)** hypoxia score and the infiltrated immune cells level. **(C)** Comparison of infiltrating immune cells between high- and low- hypoxia score groups. Up represents “positive correlation”, Down represents “negative correlation”. Sig represents “significant”, Notsig represents “not significant”.

Though DDR alteration was not associated with cervical cancer patients’ outcomes, the high hypoxia score or feature was associated with a worse outcome in the cervical patients from TCGA database (**Supplemental Figure 1**). Moreover, as hypoxia condition is usually connected with the tumor microenvironment, we evaluated the infiltrated immune cells level in cervical cancer patients with high or low hypoxia feature. Both the buffa and ragnum hypoxia scores were significantly associated with a decreasing level of CD8 positive T cells, activated mast cells but a higher level of resting mast cells and M0 macrophages (**Figures 6A**–**C**). Moreover, there is a significant abundance of NK cell resting, mast cell resting, and M0 macrophage in patients with high ragnum hypoxia score, while a significant abundance of CD8+ T cells, NK cell activated, mast cell activated, and M2 macrophage in patients with low ragnum hypoxia score (p <0.05, **Figure 6C**).

### Hub Gene(s) Identification

**Figure 7:**
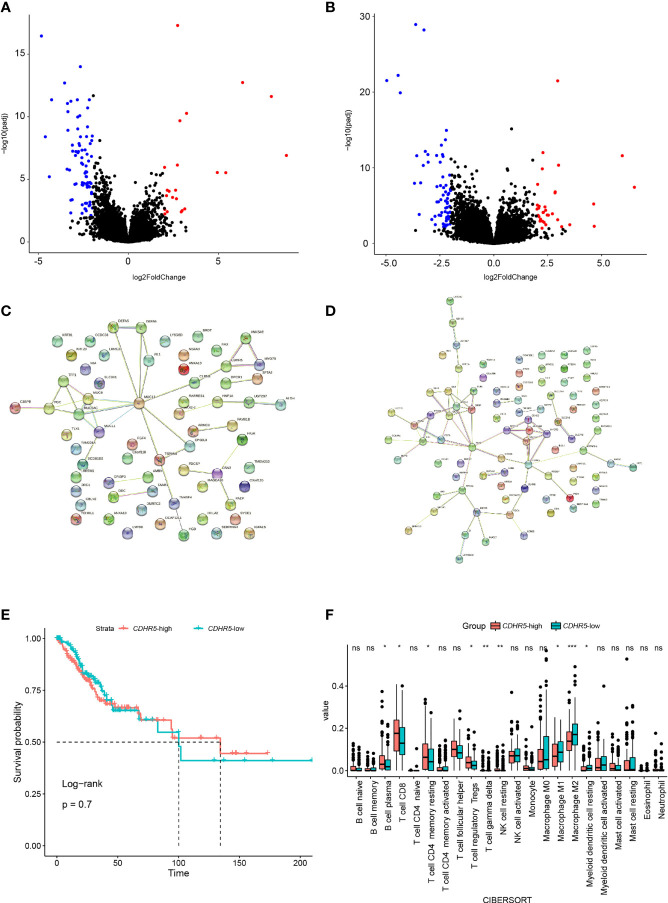
DEGs analysis between samples with and without DDR alteration **(A)**, and samples with high and low hypoxia scores **(B)**, respectively. The protein–protein interactions (PPI) network of DDR **(C)** and hypoxia score **(D)**, respectively. **(E)** The overall survival analysis between the groups with high or low expression of hub gene *CDHR5*. **(F)** Analysis of tumor infiltrated lymphocytes in cervical cancer samples with high and low *CDHR5* expression. *p < 0.05, **p < 0.01, ***p < 0.001, ns, not significant.

We conducted DEGs analysis between samples with and without DDR alteration in the TCGA cohort (**Figure 7A**), and samples with high and low hypoxia scores (**Figure 7B**), respectively. Notably, there were only three genes were identified in both the DDRmt and high hypoxia groups, namely, *CDHR5*, *MYO7B*, and *ANKS4B* (adjust p <0.01, **Supplemental Table 1**), which were all downregulated. The protein–protein interactions (PPI) network of DDR and hypoxia score was constructed by the STRING database, and hub genes were selected from the PPI network by using Maximal Clique Centrality algorithm of CytoHubba plugin, respectively (**Figures 7C, D**). The top 10 high-scored hub genes were selected, but only one gene (*CDHR5*) was shared by the two PPI network. The expression of *CDHR5* was not associated with cervical cancer patients’ survival (**Figure 7E**). However, a significant higher count of B cell, CD8 positive T cells, resting CD4 positive T memory cells, regulatory T cells, gamma delta T cells, and resting NK cells were presented in cervical cancer samples with high *CDHR5* expression. On the contrary, more M1 and M2 macrophage and myeloid dendritic cells were in the samples with low *CDHR5* expression (**Figure 7F**).

## Discussion

Over the past decades, the overall survival of advanced cervical carcinoma has not been strikingly improved, mainly attributing to slow drug development. Unlike ovarian carcinoma, the most prevalent genes in cervical carcinoma patients have poor relationship with any target therapy with high efficacy (5). Furthermore, the genetic feature of Chinese cervical carcinoma patients has not been clarified yet.

Initially, we found significant differences in the genetic features between our cohort and the TCGA database, namely, a different prevalence of *KMT2C*, *RET*, and *TP53*. The frequency of *TP53* in our cohort was nearly equivalent with previous result in a 32-patient cohort (15.50% vs 15.60%, p >0.05) (17), both of which were more prevalent among Chinese cervical patients in comparison with those (7.90%) in the Western cohort (p <0.05). Meanwhile, in a 32-sample cohort the frequency of *KMT2C* was also significantly lower than the result in the TCGA cohort (<9.00% vs 18.56%, p <0.05), but the Western groups had similar frequency of *KMT2C* (TCGA cohort vs 182-patient cohort: 18.56 vs 16.00%, p >0.05) (26). Of note, the prevalence of *RET* in Chinses cohort was first found in the present study. Our study revealed the different genetic profiles of cervical cancer patients with different genetic backgrounds. However, the prevalence of the most recurrent genes and actionable genes were similar, and notably, over 40% of investigated Chinese and Western cervical cancers patients harbored at least one actionable genomic alteration, which was also close to the previous findings of Zehir et al. (6). The most prevalent actionable alterations were in PI3K signaling pathway, especially for *PIK3CA* and *PTEN*. A recent study also demonstrated the high prevalence of *PIK3CA* alterations in cervical carcinoma patients with 31.30% altered samples, suggesting the promising targeted therapy with related PI3K or mTOR inhibitor (27). However, only limited evidences supported the correlation of *PIK3CA* alterations with the response to the mTOR inhibitors in cervical carcinoma (28). Besides, *ERBB2* is widely altered in solid tumors, especially breast and gastric cancers. Previous studies found that nearly 5.5 and 3.15% of Western and Chinese invasive cervical carcinoma patients had *ERBB2* alterations, which were associated with a worse prognosis (29, 30). In our study, we found 9.30% of the patients had *ERBB2* alterations, but 6.97% had oncogenic alterations, including 6 gains of function missense variants and 3 of amplification. Interestingly, all the oncogenic *ERBB2* alterations were only identified in the tissue samples. Early research on patient-derived xenograft derived from the cervical carcinoma patients found anti-HER2 therapy, the combination of trastuzumab and lapatinib inhibited tumor growth. Neratinib, an ERBB2 inhibitor, showed a confirmed objective response rate of 25% and progression-free survival of 7.0 months in 10 cervical carcinoma patients from the phase 2 SUMMIT basket trial (31). In addition, 10.88% of Chinese cervical carcinoma patients in our cohort were identified to harbor functional DNA damage repair alterations, similar to the prevalence in Western patients (16.15% in the TCGA cohort and 13.2% in another cohort with 824 Western cervical patients) (32). In the past decades, PARP inhibitors have been the promising targeted therapies for pan-cancers, especially for those with homology recombination deficiency. Though they have made remarkable progress in multiple solid tumors, namely, ovarian, breast, pancreatic, and prostate carcinoma, results of the efficacy of PARP inhibitors in cervical cancer are still quite poor. One study analyzed the combination of chemotherapy (paclitaxel and cisplatin) with PARP inhibitor (Veliparib) in 34 biomarker-unselected persistent or recurrent cervical carcinoma patients, showing a promising ORR of 34%, and the median PFS and OS were 6.2 and 14.5 months, respectively (33). Enlightened by the results of trails on biomarker-guided match-therapy (34), it would be recommended that these cervical cancer patients with actionable alterations in our cohort could try the matched therapy when they progressed following prior treatment or without satisfactory alternative standard treatment options.

In the present study, it was found that DDR alteration was positively correlated with the hypoxia score, especially for the deleterious DDR alteration indicating the higher hypoxia score. In addition, both the buffa and ragnum hypoxia scores, described in previous studies (22, 23), were negatively implicated with the level of CD8+ T cells which play a pivotal role in cancer immunity and are associated with a better response in patients receiving ICIs (35). The immune checkpoint inhibitors are promising treatments for various advanced cancers. FDA had approved pembrolizumab for treating patients with recurrent or metastatic cervical cancer based on the phase II KEYNOTE-158 study, though its objective response rate (ORR) was only 12.2% (4). Given the limited response rate of anti-PD-1 therapy, it is vital to identify robust biomarkers for distinguishing cervical patients who may benefit from ICIs treatment. DDR alteration was widely suggested as an effective biomarker for predicting the potential responder in multiple types of cancer, including lung, bladder, and renal cell carcinoma (36–38). Furthermore, DDR alteration may lead to genomic instability, namely, mismatch instability and chromosomal rearrangements, and further affects the tumor immune microenvironment by activating of T cells and adaptative immune system (39). However, there was no study revealing the relationship among DDR alteration, tumor microenvironment, and ICIs efficacy in cervical cancer. Our study is the first one suggesting that although DDR alteration was associated with a higher TMB value, and it was also positively related to increasing hypoxia feature, which may reshape the immune suppressive tumor microenvironment. DDR alterations, regardless of their specific function, were positively associated with both the higher hypoxia score and hypoxia feature in cervical cancer patients. Previous studies have suggested a complex relationship between hypoxia and DDR function, revealing a multifaceted regulatory role of hypoxia for DDR (40). For chronic tumor hypoxia, it downregulated most DDR pathways to silence their function in maintain genomic stability. Tumor hypoxia is not only associated with the development of malignancy and therapeutic resistance as an indicator for poor outcomes but also serves a vital determinant of tumor microenvironment (41). Previous studies also demonstrated that hypoxia could suppress the NK cell function, affect the contents of effective and regulatory T cells, and promote the polarization of macrophages to M2, a immunosuppressive phenotype (42). Thus, it could be suggested that DDR alteration could not function as a robust determinant for predicting the efficacy of ICIs in cervical cancer patients as other types of cancers, which need to be further verified.

Furthermore, we identified *CDHR5* as the significant hub gene solely related to both DDR alteration and hypoxia score. This gene belongs to the superfamily of cadherin, and participates in multiple physical processes including cell adhesion and branching morphogenesis of organs (43). Previous studies have suggested controversial roles of *CDHR5* in the cancer progression in different cancer types, but according to the decreased expression level in tumor tissues than the adjacent non-tumor tissues, it’s more likely to function as tumor suppressor (43–46). Its decreased expression in the tumor was associated with hypermethylation and transcriptional regulation. Though Beck and his colleagues found the negative correlation between *CDHR5* and DNA replication and repair (44), the explicit relationship between *CDHR5* and DDR or hypoxia has not been established yet. This is the first study that suggested the negative correlation between *CDHR5* and DDR or hypoxia in the cervical cancer, which merited further study.

The work presented here has several limitations. Firstly, it is limited by the sample size to comprehensively understand the genetic profiling of Chinese cervical cancer patients, and further study with a larger sample size is required to fully evaluate the findings. Secondly, we just investigated the potential correlation between DDR alteration, hypoxia feature, and tumor microenvironment, but whether the efficacy of ICIs in cervical cancer patients with or without DDR alteration is different merits further study.

## Conclusions

In summary, this study provided a comprehensive analysis of genomic alterations in Chinese patients with cervical cancer. Genomic profiling of Chinese patients uncovered a unique genomic feature and widely prevalent actionable variants, especially in PI3K and DDR pathways, which could guide clinical management in future. Moreover, we found the association between DDR alteration, hypoxia feature, and tumor microenvironment in cervical cancer, namely, the negatively regulated hub gene *CDHR5*, suggesting that DDR alteration(s) could not function as a robust predictor of ICIs in cervical cancer patients.

## Data Availability Statement

The original contributions presented in the study are included in the article/**Supplementary Material**. Further inquiries can be directed to the corresponding author.

## Ethics Statement

The studies involving human participants were reviewed and approved by the Fudan University Cancer Center Ethics Committee. The patients/participants provided their written informed consent to participate in this study.

## Author Contributions

JL, HW and X-LZ proposed the design of this study. HW and Q-HG collected samples and conducted data analysis. JL, HW, Q-HG and X-LZ wrote the manuscript draft. X-HW and JL revised the manuscript. All authors contributed to the article and approved the submitted version.

## Funding

This study was supported by the Female Tumor Project of Shanghai Key Clinical Specialty from Shanghai Municipal Health Commission (No. SHSLCZDZK06301).

## Conflict of Interest

The authors declare that the research was conducted in the absence of any commercial or financial relationships that could be construed as a potential conflict of interest.

## Publisher’s Note

All claims expressed in this article are solely those of the authors and do not necessarily represent those of their affiliated organizations, or those of the publisher, the editors and the reviewers. Any product that may be evaluated in this article, or claim that may be made by its manufacturer, is not guaranteed or endorsed by the publisher.
