# Supplementary material for: Genomic Profiling of Chinese Cervical Cancer Patients Reveals Prevalence of DNA Damage Repair Gene Alterations and Related Hypoxia Feature
Source: Front Oncol. 2022 Jan 7;11:792003. doi: 10.3389/fonc.2021.792003 (PMC8782566; doi:10.3389/fonc.2021.792003)
Supplement: Supplementary file 2 [file Image_1.pdf]

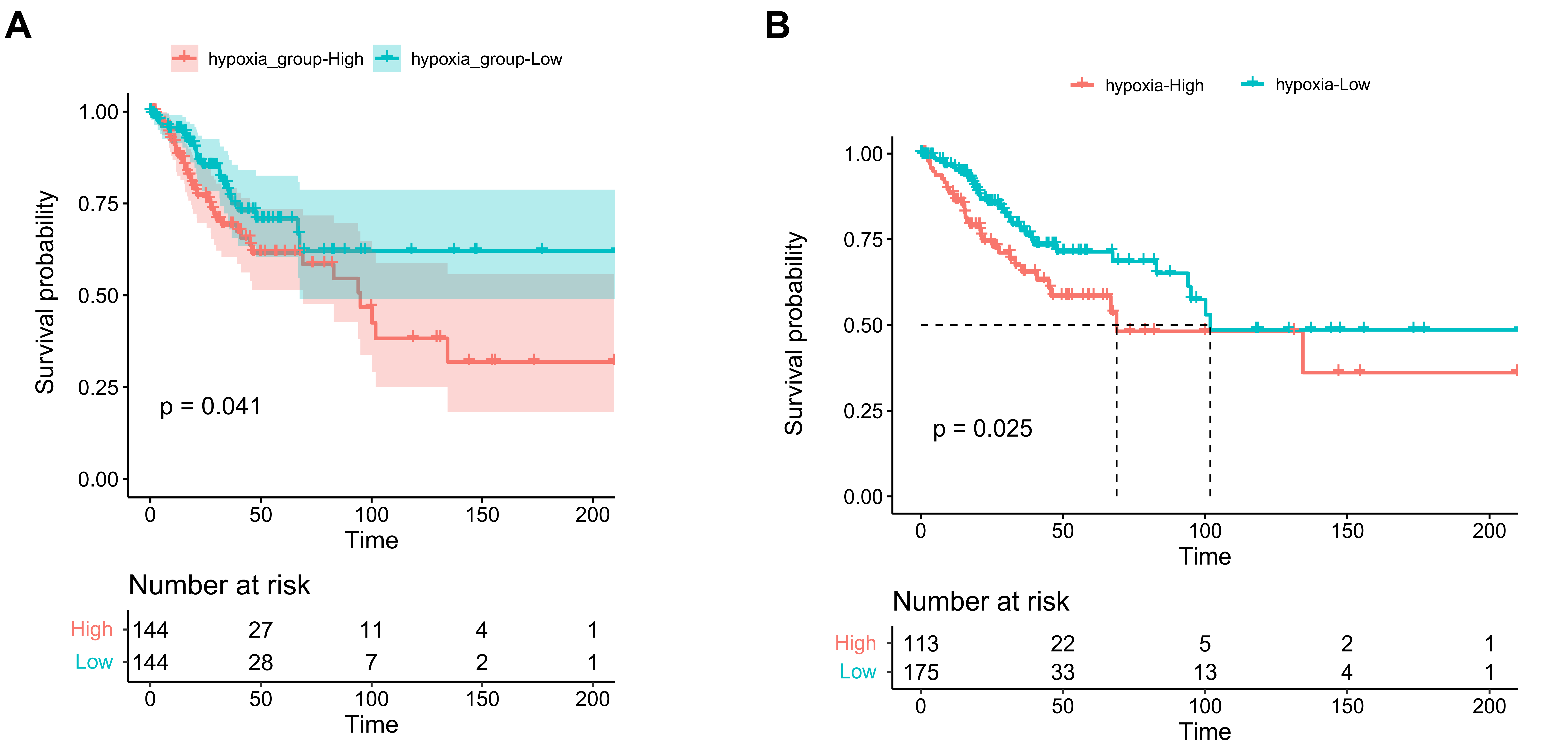

Supplementary Figure 1. A. The Kaplan-Meier curve of cervical patients who were grouped according to the analysis of hypoxia gene set. **B.** The Kaplan-Meier curve of cervical patients who were grouped by the different hypoxia score
